# Vasorelaxant Effect of Osterici Radix Ethanol Extract on Rat Aortic Rings

**DOI:** 10.1155/2013/350964

**Published:** 2013-09-24

**Authors:** Kyungjin Lee, Geunyong Park, Inhye Ham, Gabsik Yang, Mihwa Lee, Youngmin Bu, Hocheol Kim, Ho-Young Choi

**Affiliations:** Department of Herbology, College of Korean Medicine, Kyung Hee University, 26 Kyungheedae-ro, Dongdaemun-gu, Seoul 130-701, Republic of Korea

## Abstract

The root of *Ostericum koreanum* Maximowicz has been used as a traditional medicine called “Kanghwal” in Korea (or “Qianghuo” in China). The purpose of this study was to investigate the vasorelaxant activity and mechanism of action of an ethanol extract of the *O. koreanum* root (EOK). We used isolated rat aortic rings to assess the effects of EOK on various vasorelaxant or vasoconstriction factors. EOK induced vasorelaxation in phenylephrine hydrochloride (PE) or KCl precontracted aortic rings in a concentration-dependent manner. However, the vasorelaxant effects of EOK on endothelium-intact aortic rings were reduced by pretreatment with L-NAME or methylene blue. In Ca^2+^-free Krebs-Henseleit solution, pretreatment with EOK (0.3 mg/mL) completely inhibited PE-induced constriction. In addition, EOK (0.3 mg/mL) also completely inhibited vasoconstriction induced by supplemental Ca^2+^ in aortic rings that were precontracted with PE or KCl. Furthermore, the EOK-induced vasorelaxation in PE-contracted aortic rings was inhibited by preincubation with nifedipine. These results indicate that the vasorelaxant effects of EOK are responsible for the induction of NO formation from L-Arg and NO-cGMP pathways, blockage of the extracellular Ca^2+^ entry via the receptor-operative Ca^2+^ channel and voltage-dependent calcium channel, and blockage of sarcoplasmic reticulum Ca^2+^ release via the inositol triphosphate pathway.

## 1. Introduction

Traditional Chinese medicine (TCM) was introduced to Korea in the 6th century [1]. Therefore, many Korean herbal medicines (KHMs) originated as traditional Chinese herbal medicines (TCHMs). However, many components of KHMs have developed independently from TCHMs because of differences in geography, climate, culture, and politics. Thus, many KHMs different from TCHMs have been used in Korean medicine clinics. Osterici Radix, the root of *Ostericum koreanum* Maximowicz (Umbelliferae), is an example of this type of herbal medicine.

The medicinal plant *O. koreanum* is a perennial herb widely distributed in Korea. The root of this plant has been used as a traditional medicine called “Kanghwal.” However, the pharmacopoeias of Korea, China, and Japan describe this plant's origin differently. The Chinese and Japanese pharmacopoeias list only *Notopterygium incisum* and *Notopterygium forbesii* as being of “Kanghwal” origin (“Qianghuo” in Chinese), while the Korean pharmacopoeia includes *O. koreanum* as also being of “Kanghwal” origin [2–4].

Notopterygii Rhizoma et Radix, the rhizome and root of *N. incisum* and *N. forbesii*, has been used in China for the treatment of colds, headache, edema, arthritis, sores, and ulcers [5]. It is reported to have multiple effects including the elimination of fever, alleviation of pain, anti-inflammation, protection against heart palpitations and myocardial ischemia, antishock, antibacterial effects [5], and vasorelaxant effects [6]. 

Osterici Radix and Notopterygii Rhizoma et Radix have been used in Korea for the treatment of colds, fever, headache, swelling, arthritis, arthralgia, rhinitis [7], and cardiovascular diseases [6]. However, most doctors of Korean medicine have been using Osterici Radix more than Notopterygii Rhizoma et Radix as “Kanghwal” in clinics. However, there are fewer pharmacological studies and clinical data for Osterici Radix than there are for Notopterygii Rhizoma et Radix. Therefore, many more pharmacological and clinical studies are needed to support the continued use of Osterici Radix as a beneficial medicine.

Osterici Radix is reported to have various pharmacological activities: antiinflammatory [8, 9], antitumor [10], antioxidant [11], antimicrobial [12], and antiasthmatic [13]. Further, the vasorelaxant effect of water extracts of Osterici Radix on 5-HT precontracted rat thoracic aorta rings has been reported [14]. However, there are no published studies on the exact mechanism underlying the vasorelaxant effects of Osterici Radix. 

In cardiovascular diseases such as stroke, headache, and hypertension, vasoreactivity is of fundamental importance because it directly influences the arteries of the circulatory system. Accordingly, many researchers have investigated the vasorelaxant effects of various herbal medicines [15–18]. 

Therefore, we designed the present study to investigate the vasorelaxant activity and mechanism of action of the ethanol extract of the *O. koreanum* root (EOK). For this purpose, we used isolated rat thoracic aorta rings to assess the effects of EOK on various vasorelaxant or vasoconstriction factors.

## 2. Materials and Methods

### 2.1. Chemicals and Reagents

Phenylephrine hydrochloride (PE), acetylcholine (Ach), ethylene glycol-bis (*β*-aminoethyl ether)-N,N,N′,N′-tetraacetic acid (EGTA), potassium chloride, calcium chloride, N*ω*-nitro-l-arginine methyl ester (l-NAME), methylene blue (MB), Y-27632, nifedipine, tetraethylammonium (TEA), glibenclamide, 4-aminopyridine (4-AP), and caffeine were purchased from Sigma Aldrich (St Louis, USA). Barium chloride was purchased from Wako (Osaka, Japan). Isoimperatorin, imperatorin, oxypeucedanin, and oxypeucedanin hydrate were purchased from the Korean Food & Drug Administration. All other reagents were of analytical purity.

### 2.2. Plant Material and Extraction

*O. koreanum* was collected in Bongwha Alpine Medicinal Plant Experiment Station, Bongwha, Gyeongbook province, Republic of Korea, in October 2008. Plant identification was performed by Professor Chang Soo Yook of Kyung Hee University. A voucher specimen (KH001) of *O. koreanum* is deposited at the College of Korean Medicine, Kyung Hee University, Seoul, Pepublic of Korea. The dried root and rhizome of *O. koreanum* (1.0 kg) were extracted separately with 100% ethanol (3 times for 2 h at 60°C) in a reflux apparatus. After reflux and filtration, extracts were evaporated using a rotary evaporator at 60°C and lyophilized to yield 165.0 g of crude extract.

### 2.3. Preparation of Rat Aortic Ring

We used male Sprague-Dawley rats (weight, 240–260 g; Narabio, Seoul, Pepublic of Korea) to examine the vasorelaxant effect of EOK. All animal procedures were conducted according to the animal welfare guidelines issued by the Kyung Hee University Institutional Animal Care and Use Committee [KHUASP (SE)-09-006]. The rats were housed under controlled conditions (22 ± 2°C; lighting, 07:00–19:00), with food and water available *ad libitum*. Rats were anesthetized by exposure to ether; the thoracic aorta was removed and immersed in Krebs-Henseleit solution [K-H solution, composition (mM): NaCl, 118.0; KCl, 4.7; MgSO_4_, 1.2; KH_2_PO_4_, 1.2; CaCl_2_, 2.5; NaHCO_3_, 25.0; and glucose, 11.1; pH 7.4], maintained at 37°C, and aerated with a mixture of 95% O_2_ and 5% CO_2_. After careful removal of the connective tissue and fat, approximately 2 mm long aortic rings were cut and suspended in organ chambers containing 10 mL K-H solution at 37°C and aerated with a mixture of 95% O_2_ and 5% CO_2_. The aortic rings were placed between 2 tungsten stirrups and connected to an isometric force transducer (Grass instrument Co., Rhode Island, USA). After incubation under no tension for 30 min, the vessel segments were allowed to equilibrate for 1 h at a resting tension of 1.0 g. During the equilibration period, the K-H solution was replaced every 20 min. Changes in tension were recorded by isometric transducers connected to a data acquisition system (PowerLab, ADI instrument Co., New South Wales, Australia). When required, the endothelium was removed by gentle rubbing of the vessel lumen with a thin cotton swab. The presence of functional endothelium was verified by the ability of Ach (10 **μ**M) to induce more than 80% relaxation of rings precontracted by PE (1 **μ**M). In endothelium-denuded rings, less than 10% relaxation due to Ach was seen. Ca^2+^-free K-H solution was prepared by omission of CaCl_2_ and addition of EGTA (1 mM).

### 2.4. Vasoactivities

In standard K-H solution, endothelium-intact aortic rings were precontracted by PE (1 **μ**M) or KCl (60 mM). After the plateau was attained, EOK was added cumulatively (0.03–1.0 mg/mL). The vasorelaxant effect on the aortic rings was calculated as a percentage of contraction in response to PE or KCl. To determine whether the arterial endothelium pathways were involved in EOK-induced vasorelaxation, endothelium-intact aortic rings were preincubated with l-NAME (10 **μ**M) or MB (10 **μ**M) for 20 min before contraction by PE (1 **μ**M) treatment. The relaxant effects of EOK on the aortic rings were compared with the control (not treated with l-NAME or MB). To investigate the effect of EOK on extracellular Ca^2+^-induced contraction, we investigated the contractile responses induced by CaCl_2_ (0.3–10 mM) addition on aortic rings pre-contracted by PE (1 *μ*M) or KCl (60 mM) in Ca^2+^-free K-H solution in the absence (control) and presence of a 10 min pre-incubation of EOK (0.3 mg/mL). CaCl_2_-induced contractile responses were compared in the absence (control) and presence of EOK. To investigate the effect of EOK on L-type voltage-dependent calcium channel, K^+^ channels, and Rho-kinase pathway, aortic rings were preincubated with nifedipine, 4-AP, glibenclamide, TEA, or Y-27632 (1 **μ**M) for 20 min before the addition of PE (1 **μ**M). After the plateau was attained, EOK was added cumulatively (0.01–0.8 mg/mL). The vasorelaxant effects on the aortic rings were calculated as a percentage of contraction in response to PE. To investigate the effect of EOK on intracellular Ca^2+^ release from sarcoplasmic reticulum- (SR-) induced contraction, we investigated the contractile responses induced by PE (1 *μ*M) or caffeine (5 mM) on endothelium-denuded aortic rings in Ca^2+^-free K-H solution in the absence (control) and presence of a 10 min pre-incubation of EOK (0.3 mg/mL). Contractile responses induced by PE or caffeine were compared in the absence (control) and presence of EOK.

### 2.5. Qualitative and Quantitative HPLC Analysis of Standard Materials in EOK

Accurately weighted EOK (100.0 mg) was dissolved in 10 mL of methanol (HPLC reagent, J.T.Baker Co. Ltd, USA). And then it was filtered through a syringe filter (13 mm diameter with 0.45 **μ**m pore size, Waters, MA, USA). Isoimperatorin, imperatorin, and oxypeucedanin hydrate were used as standard materials for the qualitative analysis of EOK. They were serially diluted (25, 50, 100, and 200 *μ*g/mL), and HPLC chromatograms were obtained. The relationship between the concentration and the peak area was measured using the minimum square method (*R* ^2^ value). The HPLC chromatograms were obtained from Gilson System equipped with a 234 autoinjector, a UV/VIS-155 detector, and a 321 HPLC Pump (Gilson, WI, USA). A Luna 4.60 × 250 mm C18 reversed-phase column with 5 **μ**m particles (Phenomenex, CA, USA) was used. Chromatographic separation was carried out using an isocratic solvent with acetonitrile (HPLC grade, J.T.Baker Co. LTD., U.S.A) water mixture in the ratio of 30 : 70 (v/v). The column eluent was monitored at UV 254 nm, following which all solvents were degassed with a micromembrane filter (PTFE, Advantec, Tokyo, Japan). Chromatography was performed at room temperature at a flow rate of 1.0 mL/min, and 10 *μ*L was analyzed for 20 min. The quantity of the EOK standards was expressed as follows: the amount (mg) of standard material = the quantitative amount (mg) of standard materials × *A*_T_/*A*_S_/*n* (*n* = 3; *A*_T_ = the peak area of the test sample containing the standard; *A*_S_ = the peak area of the standard). 

### 2.6. Statistical Analysis

 Data were expressed as mean ± standard error of mean (SEM). Statistical comparisons were made using Student's *t*-test or one-way analysis of variance (ANOVA) followed by the Tukey's post hoc test. All statistical analyses were performed using SPSS v.13.0 statistical analysis software (SPSS Inc., USA). *P* values less than 0.05 were considered statistically significant.

## 3. Results

### 3.1. Effect of EOK on PE- or KCl-Induced Contraction

**Figure 1:**
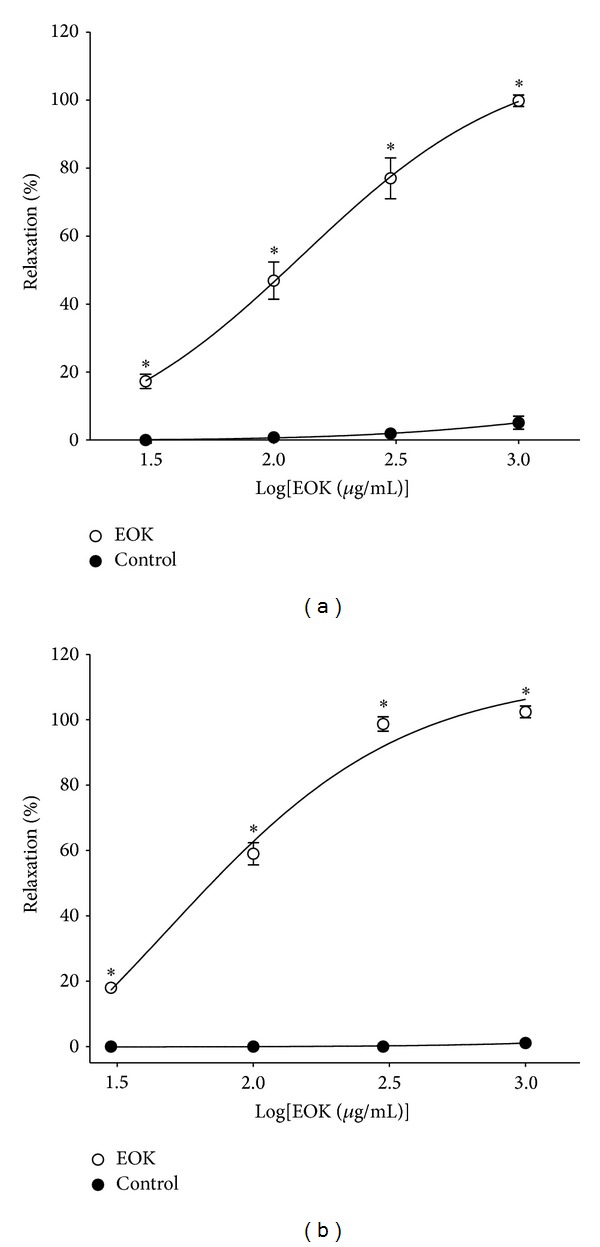
Concentration-dependent relaxant effects of EOK (0.03–1.0 mg/mL) on phenylephrine (PE, 1 **μ**M) (a) or KCl (60 mM) (b) precontracted rat aortic rings. Control groups were not treated with EOK. The relaxant effects of EOK were calculated as a percentage of the contraction in response to PE or KCl. Values are expressed as mean ± SEM (*n* = 4). *P < 0.05 versus  control.

 EOK (0.03–1.0 mg/mL) relaxed PE (1 *μ*M) or KCl (60 mM) precontracted rings in a concentration-dependent manner. The maximal relaxant effects were up to 99.8 ± 1.7% and 102.4 ± 3.6% at the concentration of 1.0 mg/mL, respectively (Figure 1). 

### 3.2. Vasorelaxant Effects of EOK on Endothelium-Dependent Pathways

**Figure 2:**
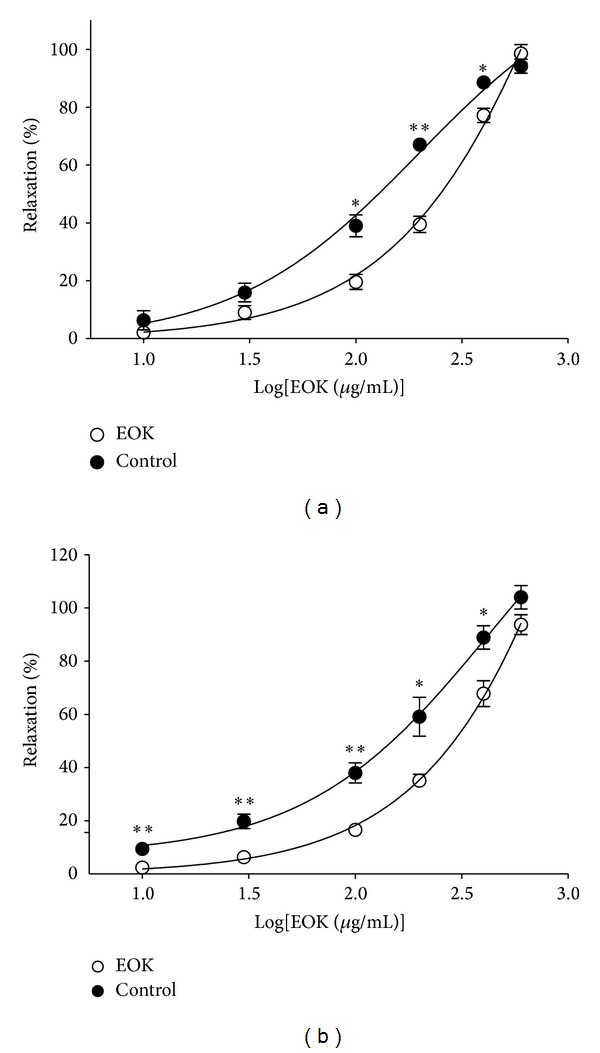
Relaxation responses induced by EOK (10–600 **μ**g/mL) in endothelium-intact rat aortic rings precontracted with phenylephrine (PE, 1 **μ**M) in the presence or absence (control) of N*ω*-nitro-l-arginine methyl ester (l-NAME, 10 **μ**M) (a) or methylene blue (MB, 10 **μ**M) (b) in Krebs-Henseleit solution. The relaxant effects of EOK on isolated rat aortic rings were calculated as a percentage of the contraction in response to PE. Control groups were not treated with l-NAME or MB. Values are expressed as mean ± SEM (*n* = 4). *P < 0.05 and **P < 0.01 versus control.

 Preincubation with l-NAME (10 *μ*M) decreased EOK- (0.1, 0.2, and 0.4 mg/mL) induced relaxation of endothelium-intact aortic rings precontracted by PE (1 *μ*M) treatment (Figure 2(a)). And, preincubation with MB (10 *μ*M) also decreased EOK- (0.01–0.4 mg/mL) induced relaxation of endothelium-intact aortic rings (Figure 2(b)). 

### 3.3. Effect of EOK on Extracellular Ca^2+^-Induced Contraction

**Figure 3:**
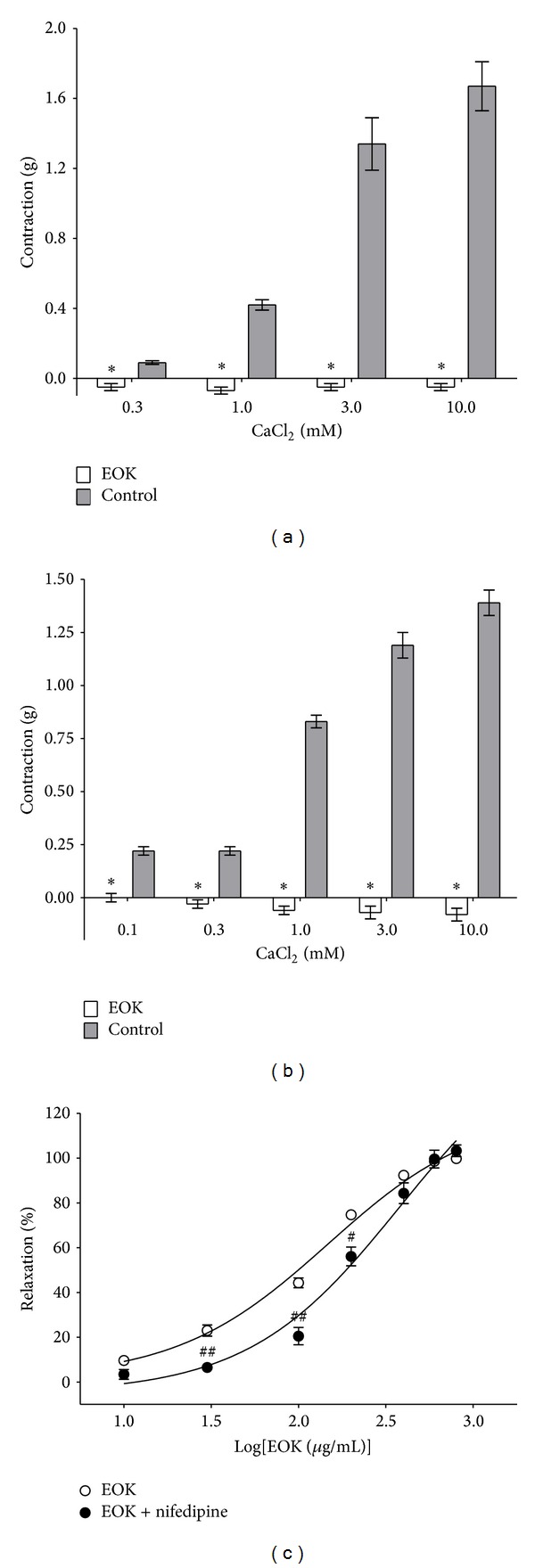
Inhibitory effect of EOK (0.3 mg/mL) on the contraction induced by extracellular Ca^2+^ addition (0.3–10 mM) in rat aortic rings precontracted with phenylephrine (PE, 1 *μ*M) (a) or KCl (60 mM) (b) in Ca^2+^-free Krebs-Henseleit solution and vasorelaxant responses induced by EOK (0.01–0.8 mg/mL) in rat aortic rings precontracted with PE (1 **μ**M) in the presence or absence of nifedipine (10 **μ**M). Values are expressed as mean ± SEM (*n* = 4). *P < 0.05 versus control. ^#^ *P* < 0.05 and ^##^ *P* < 0.01 versus absence of nifedipine group.

 To investigate the effects of EOK on the receptor-operative Ca^2+^ channel (ROCC) pathway, PE (1 **μ**M) was applied to induce stable contraction. CaCl_2_ (0.3–10 mM) was then added cumulatively to induce a progressive increase in contraction of the aortic rings. EOK (0.3 mg/mL) preincubation significantly inhibited the contraction induced by extracellular CaCl_2_ (0.3–10 mM) compared with the control group. And the contraction was decreased to −0.05 ± 0.02 g, −0.07 ± 0.02 g, −0.05 ± 0.02 g, and −0.05 ± 0.02 g, respectively (versus control group 0.09 ± 0.01 g, 0.42 ± 0.03 g, 1.34 ± 0.15 g, and 1.67 ± 0.14 g) (Figure 3(a)). To investigate the voltage-dependent calcium channel (VDCC) pathway, KCl (60 mM) was applied to induce a stable contraction. EOK (0.3 mg/mL) preincubation also significantly inhibited extracellular CaCl_2_-induced (0.1–10 mM) contraction compared with the control group. Contraction decreased to 0.00 ± 0.02 g, −0.03 ± 0.02 g, −0.06 ± 0.02 g, −0.07 ± 0.03 g, and −0.08 ±0.03 g, respectively (versus control group 0.22 ± 0.02 g, 0.22 ± 0.02 g, 0.83 ± 0.03 g, 1.19 ± 0.06 g, and 1.39 ± 0.06 g) (Figure 3(b)). In addition, preincubation with nifedipine (10 **μ**M) for 20 min inhibited EOK-induced vasorelaxation on PE-contracted aortic rings (Figure 3(c)). 

### 3.4. Effect of EOK on the Sarcoplasmic Reticulum Calcium Release Induced by PE or Caffeine

**Figure 4:**
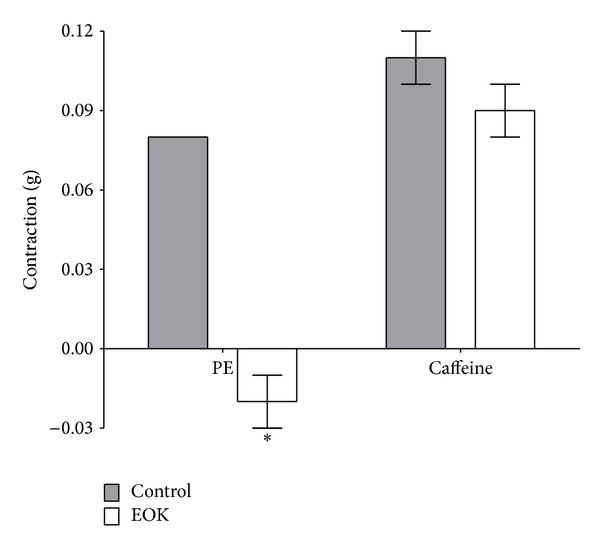
Inhibitory responses of EOK (0.3 mg/mL) preincubation on phenylephrine- (PE-, 1 **μ**M)- or caffeine- (5 mM) induced contractions of rat aortic rings. Values are expressed as mean ± SEM (*n* = 4). Control groups were not treated with EOK. *P < 0.05 versus control.

In the Ca^2+^-free K-H solution, preincubation with EOK (0.3 mg/mL) for 10 min completely inhibited PE-induced (1 **μ**M) contraction. However, preincubation with EOK (0.3 mg/mL) for 10 min did not alter caffeine-induced (5 mM) contraction (Figure 4).

### 3.5. Effect of EOK on the Rho-Kinase Pathway

**Figure 5:**
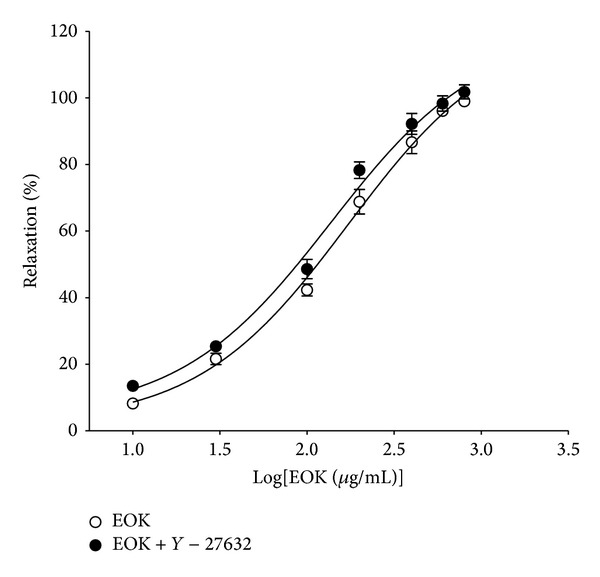
Vasorelaxant responses induced by EOK (0.01–0.8 mg/mL) in rat aortic rings precontracted with phenylephrine (1 **μ**M) in the presence or absence of Y-27632 (1 **μ**M). Values are expressed as mean ± SEM (*n* = 4).

Preincubation with Y-27632 (1 **μ**M) for 20 min did not alter EOK-induced vasorelaxation on PE-contracted aortic rings (Figure 5). 

### 3.6. Effect of EOK on the K^+^ Channels

**Figure 6:**
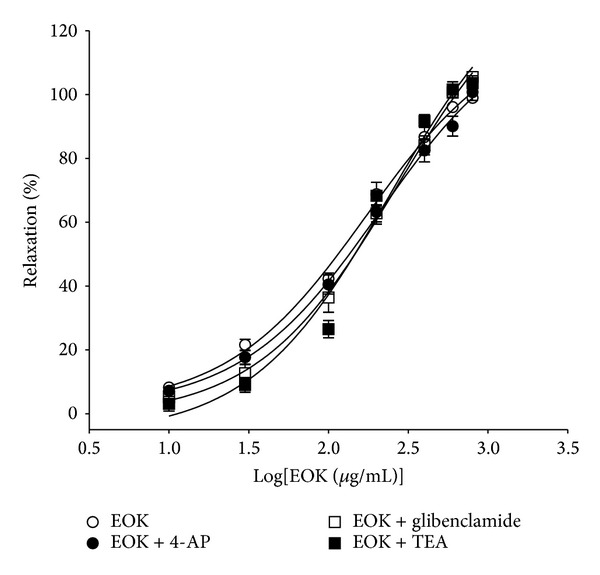
Vasorelaxant responses induced by EOK (0.01–0.8 mg/mL) in rat aortic rings precontracted with phenylephrine (1 **μ**M) in the presence or absence of 4-aminopyridine (4-AP, 1 mM), glibenclamide (10 **μ**M), or tetraethylammonium (TEA, 5 mM). Values are expressed as mean ± SEM (*n* = 4).

The vasorelaxant effects of EOK on PE (1 **μ**M) precontracted aortic rings were not altered by preincubation of the rings with various K^+^ channel blockers, including 4-AP (1 mM), glibenclamide (10 **μ**M), or TEA (5 mM) (Figure 6). 

### 3.7. Qualitative and Quantitative HPLC Analysis of Standard Materials in EOK

**Figure 7:**
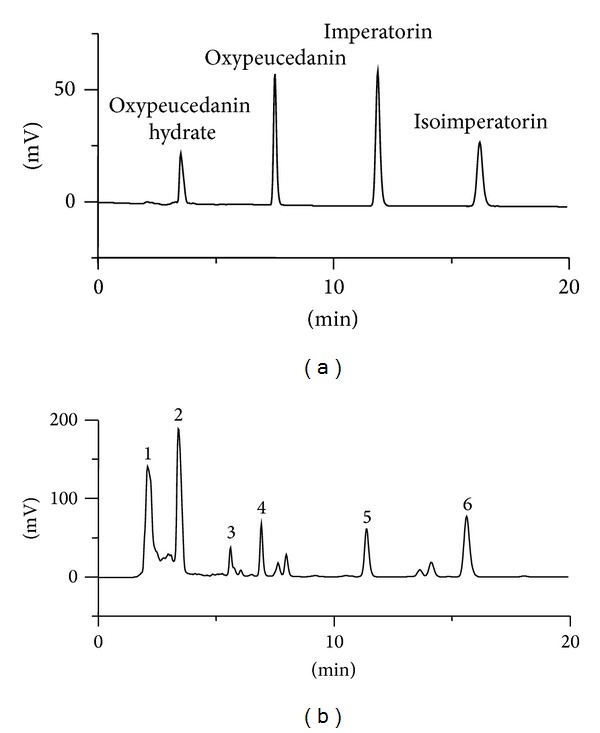
Qualitative and quantitative HPLC analysis of standard materials in EOK. The retention time of the peak 1, peak 2 (oxypeucedanin hydrate), peak 3, peak 4, peak 5 (imperatorin), and peak 6 (isoimperatorin) was 2.04 min, 3.26 min, 5.35 min, 6.57 min, 10.99 min, and 15.02 min, respectively.

Figure 7 depicts the 6 principal peaks detected on the EOK HPLC chromatogram; the retention times of the peaks were as follows: peak 1, 2.04 min; peak 2 (oxypeucedanin hydrate), 3.26 min; peak 3, 5.35 min; peak 4, 6.57 min; peak 5 (imperatorin), 10.99 min; and peak 6 (isoimperatorin), 15.02 min. The standard curve was calibrated by using the linear regression derived from the peak area. The regression equation (correlation coefficient, *R* ^2^) of oxypeucedanin hydrate was *y* = 201168.349*x* − 153935.071 (0.999) and exhibited good linearity. The oxypeucedanin hydrate content in EOK was 1.80 ± 0.92%. The regression equation (correlation coefficient, *R* ^2^) of imperatorin was *y* = 514722.569 + 153935.071 (0.999) and exhibited good linearity. The imperatorin content in EOK was 0.42 ± 0.02%. The regression equation (correlation coefficient, *R* ^2^) of isoimperatorin was *y* = 301715.046*x* + 32227.015 (0.998) and exhibited good linearity. The isoimperatorin content in EOK was 0.70 ± 0.02%. 

## 4. Discussion 

In the present study, we investigated the vasorelaxant effects of EOK on rat aortic rings and its related mechanisms. The vasorelaxant effects caused by EOK were both endothelium dependent and endothelium independent. And the vasorelaxant mechanisms of EOK were responsible for the induction of NO formation from l-Arg and NO-cGMP pathways, blockage of the extracellular Ca^2+^ entry via the ROCC and VDCC, and blockage of SR Ca^2+^ release via the IP_3_ pathway. 

Vascular endothelium plays an important role in vasorelaxation. NO is one of the potent vasodilators secreted from vascular endothelium. And vascular smooth muscle is relaxed via NO-cGMP pathway [19]. In the present study, the vasorelaxant effect of EOK was reduced by pretreatment with l-NAME, an inhibitor of NOS. In addition, the vasorelaxant effect of EOK was reduced by pretreatment with MB, a soluble guanylate cyclase inhibitor. These results suggested that the vasorelaxant effect of EOK is related to the induction of NO formation from l-arginine and NO-cGMP pathways. 

Vascular smooth muscle also plays an important role in vasorelaxation, which is regulated by extracellular Ca^2+^ influx via transmembrane Ca^2+^ channels and Ca^2+^ release from intracellular stores [20]. Ca^2+^ concentration in vascular cells is the most important factor of vascular contraction and relaxation. The factors that increase calcium in cells consist of stimulus by neurotransmitter, opened calcium channels by membrane potential, and calcium ion release from the SR. Ca^2+^ concentration in cells increases to combine with calmodulin, a protein that binds to calcium in cells. The bonded Ca^2+^-calmodulin activates inactive myosin light chain kinase, which then phosphorylates myosin light. The phosphorylated myosin light chain then makes a phosphorylated crossbridge with actin to produce a contraction [20].

PE (an *α*-adrenergic agonist) contracts smooth muscle cells via extracellular Ca^2+^ influx through ROCC and by internal calcium release from specific IP_3_ receptor (IP_3_R) channels in the SR membrane [21]. In Ca^2+^-free K-H solution, PE induced the contraction via the IP_3_ pathway. In the present study, pretreatment with EOK (0.3 mg/mL) for 10 min completely inhibited PE-induced (1 **μ**M) contraction. This result suggests that EOK could inhibit Ca^2+^-induced vasoconstriction from specific IP_3_R channels in the SR membrane. And EOK (0.3 mg/mL) completely inhibited vasoconstriction induced by Ca^2+^ supplementation in aortic rings that were precontracted with PE (1 **μ**M). This result suggested that EOK could inhibit vasoconstriction induced by extracellular Ca^2+^ entry via the ROCC.

KCl contracts smooth muscle cells mainly by extracellular Ca^2+^ influx through depolarization of the cell membrane and subsequent opening of VDCC [22]. EOK (0.3 mg/mL) also completely inhibited vasoconstriction induced by Ca^2+^ supplementation in aortic rings that were precontracted with KCl (60 mM). This result suggests that EOK could also inhibit vasoconstriction induced by extracellular Ca^2+^ entry via the VDCC. L-type VDCC is the major mechanism of VDCC. In the present study, EOK-induced vasorelaxation on PE-contracted aortic rings was inhibited by preincubation with nifedipine (a typical L-type VDCC blocker). This result suggested that EOK could inhibit extracellular Ca^2+^ entry via blocking L-type VDCC. 

Caffeine contracts smooth muscle cells by internal calcium release from ryanodine receptor (RyR) channels in the SR membrane [23]. EOK (0.3 mg/mL) did not inhibit caffeine-induced (5 mM) vasoconstriction, which suggested that EOK could not affect RyR channels.

Increase of K^+^ efflux in vascular smooth muscle causes membrane potential hyperpolarization [22]. Thus, the opening of K^+^ channels leads to vasorelaxation and the inhibition of K^+^ channels leads to vasoconstriction. Voltage-dependent K^+^ (K_V_) channels, Ca^2+^-activated K^+^ (BK_Ca_) channels, and ATP-sensitive K^+^ (K_ATP_) channels are well-known distinct types of K^+^ channels in vascular smooth muscle [22]. In the present study, the vasorelaxant effects of EOK were not affected by preincubation of 4-AP (K_V_ channel blocker), TEA (BK_Ca_ channel blocker), and glibenclamide (K_ATP_ channel blocker). These results indicated that the vasorelaxant effects of EOK on the rat aortic rings were not related to the opening of K^+^ channels.

Rho kinase has been identified as one of the effectors of the small GTP-binding protein Rho. And Rho/Rho-kinase-mediated pathway has been implicated in the regulation of vascular tone [24]. Y-27632 is a Rho-kinase inhibitor that blocks agonist-induced Ca^2+^ sensitization of smooth muscle [25]. In this study, Y-27632 did not significantly affect the relaxant effect of EOK. This result indicated that the relaxant effect of EOK on rat aortic ring is not related to the Rho-kinase pathway.

Osterici Radix has been reported to contain several active compounds such as oxypeucedanin hydrate [11], oxypeucedanin, imperatorin, and isoimperatorin [26]. In the present study, we identified oxypeucedanin hydrate (1.80 ± 0.92%), imperatorin (0.42 ± 0.02%), and isoimperatorin (0.70 ± 0.02%) from EOK by using HPLC analysis. Imperatorin is reported to relax rat mesenteric arteries precontracted by KCl or endothelin-1 and human omental arteries precontracted by noradrenaline and U46619 [27]. And isoimperatorin is also reported to relax rat aortic rings precontracted by PE or KCl [28]. Therefore, vasorelaxant activities of EOK might result from these active compounds. However, vasorelaxant activity of oxypeucedanin hydrate has not yet been studied, and EOK possesses many other multiple known and unknown compounds. Thus, it is necessary to find more active compounds for the evidence of vasorelaxant activities of EOK. 

 In conclusions our findings suggest that Osterici Radix might be a useful herbal medicine for treating cardiovascular diseases such as hypertension. However, more detailed mechanism studies, *in vivo* studies, and chemical analyses may be necessary to establish the efficacy of Osterici Radix for treating hypertension.
